# A Webcast of Bird Nesting as a State-of-the-Art Citizen Science

**DOI:** 10.1371/journal.pbio.2001132

**Published:** 2017-01-06

**Authors:** Markéta Zárybnická, Petr Sklenicka, Piotr Tryjanowski

**Affiliations:** 1 Faculty of Environmental Sciences, Czech University of Life Sciences Prague, Prague, Czech Republic; 2 Institute of Zoology, Poznań University of Life Sciences, Poznań, Poland

## Abstract

The quality of people’s knowledge of nature has always had a significant influence on their approach to wildlife and nature conservation. However, direct interactions of people with nature are greatly limited nowadays, especially because of urbanization and modern lifestyles. As a result, our isolation from the natural world has been growing. Here, we present an example of a state-of-the-art Citizen Science project with its educational, scientific, and popularizing benefits. We conclude that modern media and new forms of education offer an effective opportunity for inspiring children and others to have fun learning to act like scientists. This approach provides broad opportunities for developing the hitherto neglected educational potential of Citizen Science.

## Introduction

Industrialization, urbanization, and modern lifestyles have nowadays greatly reduced our direct interactions with nature. As a result, people’s knowledge of nature has become restricted. Children, in particular, are better able to identify a variety of artificial creatures and extinct animals than the common wild animals from the real, present-day world around them. They seem to learn far more about Pokémon than about nature and wildlife [1]. Our growing isolation from the natural world has direct and grave consequences for the conservation of biodiversity and for efforts to live in harmony with nature. It is necessary to develop new ways to raise the awareness of the next generation about the natural world of which they form a part.

In this sense, Citizen Science (CS) offers an interesting approach: it involves the public in authentic research alongside professionals, and thus, it can provide both educational benefits for the public and large amounts of data for researchers (e.g., [2–4]). Although CS offers a promising approach, its full potential has not yet been realized. On the one hand, CS projects have recently offered a platform for unique research in terms of the topic [5, 6] and the extent of the data [4, 7]. On the other hand, they are often in poor synergy with the education system, and they also insufficiently emphasize synergies between environmental education and science education [8]. High-quality collaboration among scientists, project organizers, government institutions, and the public is also rare [9]. Moreover, volunteers in CS projects very often gather data that is of low quality and of limited validity [10, 11], or the methodologies that are used cannot be implemented across society, because they focus on a narrow circle of volunteers or skilled amateurs. Project outputs are usually not presented clearly to the general public or even to the volunteers involved [2, 11]. Last but not least, there are poor strategies for popularizing CS and, indeed, for popularizing scientific research in general [12, 13]. As a result, current CS projects tend to have only a limited educational impact, narrow application across society, and scanty direct benefits for the general public.

Despite the intensive process of urbanization and modern lifestyles (more than 50% of the world’s human population lives in urban areas, and over 67% of humans will live in cities by 2050 [14]), many people all over the world love nature and wildlife, and they are willing to contribute actively to their conservation. Such people like and need solutions that are good for nature alone, and not those good for humans only [15]. In this light, some CS projects, such as Cornell University’s Nest Watch Program [16], which works on the basis of watchers searching actively and visiting nests, have run successfully over periods of years. A type of activity that is extended all over the world involves hanging up artificial boxes for birds and other animals, e.g., bats, snakes and mammals, that provide a wide range of opportunities for monitoring how animals breed, roost, hibernate, store food, etc. [17–20]. In recent decades, the noninvasive method of camera monitoring in bird houses has allowed animal life to be zoomed to people [21, 22]. However, the application of camera systems for animal monitoring still suffers from some limitations (e.g., insufficient data storage, limited power sources, poor light conditions, and vulnerability to inclement weather [21, 23]).

### An Example of a State-of-the-Art CS Project

**Fig 1:**
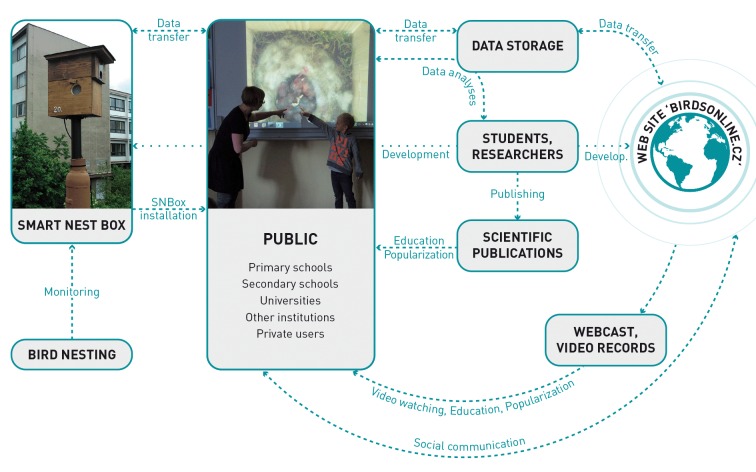
An example of a state-of-the-art Citizen Science project, with direct benefits for the public and for researchers.

The interdisciplinary BirdsOnline project, which has been developed in the Czech Republic since 2012 (Fig 1), is an example of a comprehensive project that has been dealing successfully with many of the problems mentioned above and that presents a new approach for CS in the future. The cornerstone of the project is the Smart Nest Box (SNBox), a bird box equipped with a computer, one or two cameras, an optical sensor that senses activity, temperature and light sensors, and a microphone. SNBox has been developed through interdisciplinary collaboration between biologists and technicians for monitoring the daytime and nocturnal activities of animals, and it is characterized by some specific technical features. For example, every bird’s activity is detected by a light barrier placed in the opening of the SNBox. This activates video recording for 30–120 s, depending on the user setting. The trigger speed (i.e., the time delay between disruption of the light barrier and triggering the first camera frame) has been reduced to 16 ms, and all bird activities are recorded and stored (for details, see [24]). Anyone can watch the activity in the nests through direct transmission or videos. Video streams are stored in a computer located in the box, from which they are transmitted by the user’s local Internet network to the central server. The nesting activities can be watched either in a live webcast or in videos presented with a delay of one day on a webpage accessible to the public without registration (birdsonline.cz).

The BirdsOnline project, which has been developed and tested over the last 4 years, was made accessible to the public for the first time in 2016. A set of 25 SNBoxes for monitoring songbird nesting in an urban environment and also owls nesting in a forest environment has been lent out to various state and private users. There has been enormous interest in participating in the project—within a period of 4 months, 109 new institutions were registered. Of these, 37% are primary and special schools, 18% preschools, 11% secondary and vocational training schools, 11% nongovernmental organizations (NGOs), 9% commercial companies, 6% state administration, 5% hospitals, and 2% religious institutions (S1 Data). In the period from April to July 2016, 92% of the hanging SNBoxes (*n* = 23) were occupied, and in 36% of the boxes (*n* = 9), two or three consecutive nestings were observed. The most frequent nesters were four commonly occurring bird species active during the day (great tit [*Parus major*], Eurasian blue tit [*Cyanistes caeruleus*], common starling [*Sturnus vulgaris*], and Eurasian tree sparrow [*Passer montanus*]) and also Tengmalm’s owl [*Aegolius funereus*], which is active at night. In the first half of 2016 alone, more than 100,000 video records of bird activities from 35 nests were collected.

### Educational Impact Across Society

**Fig 2:**
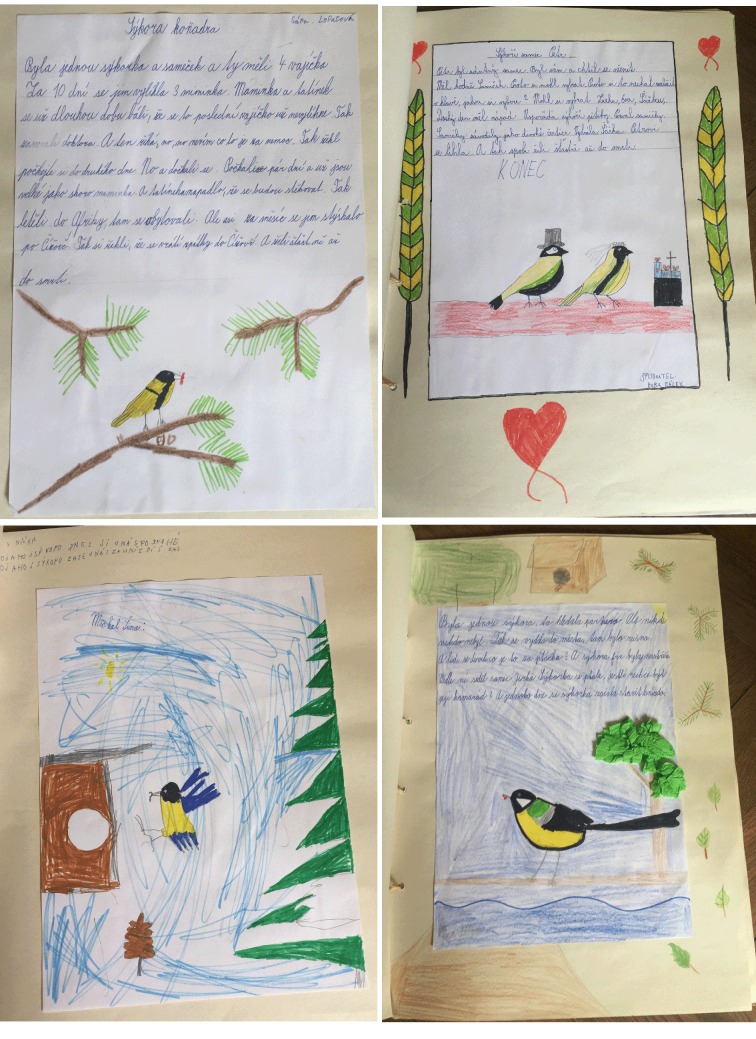
An example of a “bird book” created by schoolchildren.

Participants are able to engage actively in the BirdsOnline project in various ways. Primary, secondary, and special schools that have hung an SNBox in their own schoolyard or garden mainly use direct observations of nesting on interactive whiteboards and introduce video inputs directly into lessons on the environment and on biology, as a stimulating visual teaching aid. Schoolchildren have strengthened their relationship with the environment and with the bird life that they have documented via, for example, creative writing and painting (Fig 2). Through tests of the biological knowledge of 9- to 10-year-old schoolchildren (*n* = 53), we found differences between students’ knowledge before and after bird monitoring: 68% before and 91% after were able to identify bird species (McNemar, χ2 = 9.6, *p* = 0.002), 46% before and 89% after knew the structure of the nest material (χ2 = 22.0, *p* < 0.001), 29% before and 88% after knew the duration of the breeding period (χ2 = 31.0, *p* < 0.001), and 32% before and 93% after were able to identify the structure of the birds’ diet (χ2 = 28.7, *p* < 0.001, S1 Text, S2 Data).

**Fig 3:**
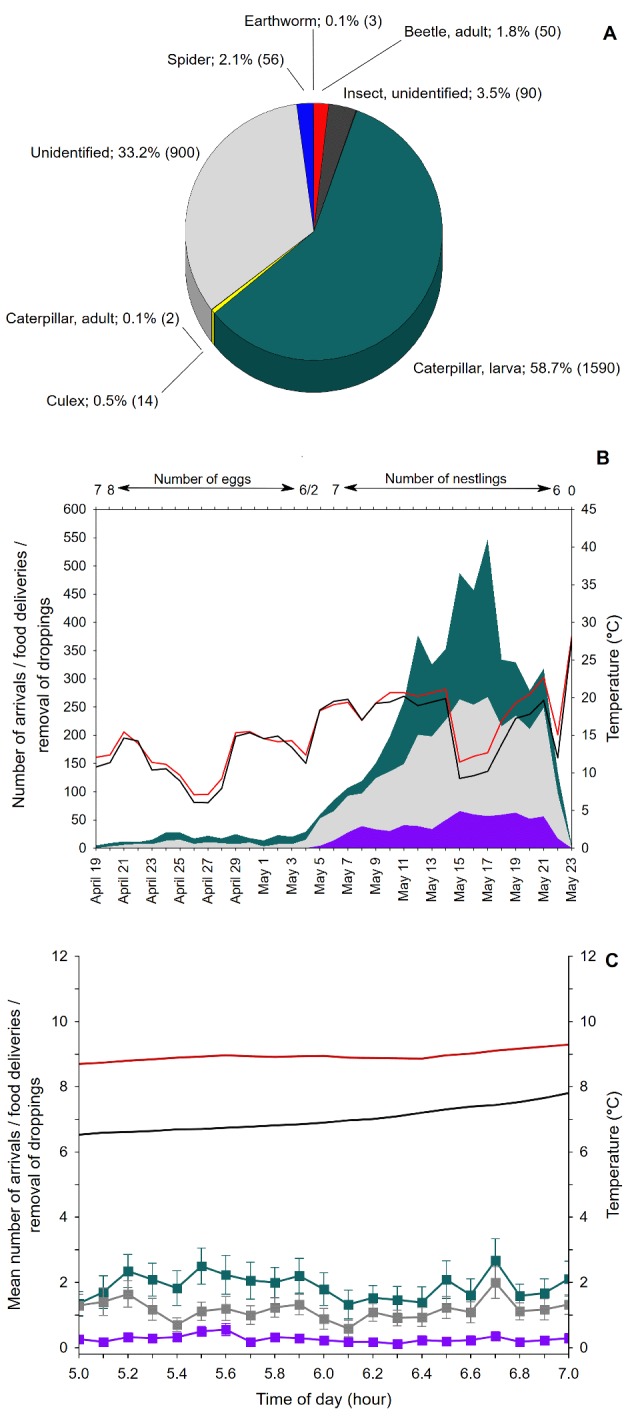
An example of data collected from the nest of a great tit (*P*. *major*) during the nesting period (i.e., from April 19 to May 23) documenting (A) the structure of the diet delivered by tit parents to nestlings (the proportions and the number of items are shown); (B) the total number of arrivals (green area), feeding deliveries (grey area), and the removal of droppings (violet area) by bird parents, including the mean daily temperature outside the nest box (black line) and inside the nest box (red line); (C) the mean daily number of arrivals, feeding deliveries, and removals of droppings by bird parents in a 6-minute period between 5 am and 7 am, including the mean daily temperature outside and inside the nest box (box: mean; whiskers: SE). For detailed information, see S2 Text and S4 Data.

We suggest that there are two important aspects of our project that strengthen the educational impact on students. Firstly, anonymous textbook information is supplemented by or replaced by a personal relationship with a place and with particular bird individuals. For example, children can observe what kind of food is gathered by “their” tits in “their” schoolyard (Fig 3A). Secondly, they have an opportunity to analyse “their” biological data in the context of all the data that have been gathered and to compare the results for “their” nesting pair with the results for other pairs. The results show children the variability of biological data and can arouse curiosity about the causes of this variability. In this light, with his groundbreaking schoolbook *Orbis Pictus*, 17th-century Czech educator J. A. Comenius encouraged learning through play rather than through performing tedious tasks. The 21st-century Czech BirdsOnline project continues in the same spirit, inspiring children and others to have fun learning to act like scientists.

Apart from gaining biological knowledge and deepening their relationship with their environment, students from secondary schools can develop their technical skills. For example, students at secondary school use the recorded video data to produce videos on tit nesting, while students in the higher classes of vocational training schools make wooden boxes with built-in structures for technical devices (S1 Fig). During this process, students develop their technical skills in machining and material processing and also learn how to process project documentation.

Private users usually observe nesting online for relaxation, and they may also participate directly in analysing the videos. These participants are motivated to get involved in monitoring and evaluation through their interest in the subject and through lifelong learning courses, or they are interested in being participants in a popular education research project (see the reference provided by a private user in a Reuters report). Public and private institutions and enterprises want to accentuate environmentally responsible corporate behaviour. For example, people from the Ministry of the Environment of the Czech Republic installed an SNBox on the roof of their building, in a highly urban environment, where great tit nesting was observed, and they later issued a press release presenting their box and their birds.

A university team manages the project and makes effective use of the inputs and outputs in the educational process. Master's and doctoral projects have been investigating SNBox design and construction and remote data transmission and have worked on creating a web environment (e.g., [25]). The data obtained on bird behaviour are now being used in bachelor’s, master's, and doctoral theses (e.g., [26]).

### Scientific Impact

The BirdsOnline project has presented a range of high-quality scientific information accompanied by very attractive video records that are accessible to everyone. During a 4-month period in 2016, we documented a range of unique information on bird nesting, including interspecies competition—for example, predation of a Tengmalm’s owl nest by a pine marten *Martes martes*; predation of a starling nest by a house sparrow; and great tit chicks dying from an infection caused by parasites of *Ornithonyssus* spp. (see S1 Video). We also showed starling courtship accompanied by singing and nest decorating, feeding of great tit chicks, and parents removing droppings and cleaning the nest. The scientific data can be used to evaluate reproductive activities and reproductive success, parental care, and diet structure, depending on environmental factors (e.g., temperature, light intensity, altitude, time-of-day, different habitat types). Everyone has an opportunity to analyse basic biological data, e.g., bird activities and behaviour, including food delivery and removal of droppings, in relation to the time of day and the breeding season and also in relation to environmental conditions, such as temperature and light intensity (Fig 3). The data collected by SNBox on Tengmalm’s owl nesting have been of high enough quality to be published in more than ten scientific journals (e.g., [27–30]). The BirdsOnline project also has the potential to collaborate with research teams across geographic areas, and it can focus on variable animal taxa. Last but not least, a wide spectrum of video records of animal behaviour provides a great database for use in computational analysis. In particular, the latest methodological directions offer automated approaches from machine vision and learning to behavioural analysis. These approaches have the ability to analyse datasets that are orders of magnitude larger, to discover features that humans cannot, and to provide a vocabulary for discussing and describing behaviour that is consistent across laboratories and even across organisms [31].

### Popularization Impact

The audience of the BirdsOnline project shares new biologically oriented information via social networks. Over a 4-month period, the website was visited by 20,000 users from 40 countries (data from Google Analytics, for details see S3 Data). In addition, foreign and national media, including radio stations, TV entertainment, and news programmes, presented the project as an example of effective collaboration between a research institution and the public. We suggest that clarity, simplicity, and attractiveness are features that make this CS project public friendly. In particular, we suggest that the project provides four main benefits: a time-efficient approach for the public, due to the use of high technology; direct contact between man and nature, due to the installation of SNBoxes on private patches; opportunities to implement the project in an urban environment; and the possibility (but not the necessity) to share bird nesting from users’ own backyards with others through social networks. These benefits enable the scientific project to address wide audiences and to achieve more efficient learning outcomes. Apart from these benefits, we also recognize some weaknesses of the project. A major drawback is that the costs of the technologies (ca. US$600 per box) restrict the use of SNBox. These costs are mostly reimbursed by research grants and by other types of grants. The implementation of the project can also be limited by geographic conditions, by traditional ways of life, by the ways in which education is implemented, and by environmental considerations. Nevertheless, the essence of the project lies in the opportunity that it provides for anyone to watch the activity in the monitored nests without restriction. This makes the project widely implementable across society and across geographical areas.

### Education Via CS

As Balmford et al. have noted, “people care about what they know” [1]. In this sense, CS projects have enormous potential as broad-spectrum tools for public education and nature conservation. In the last two decades, the development of the Internet, social media, and mobile applications has led to a great boom in CS projects. Modern media and new forms of education offer an effective opportunity for real nature to compete with fictitious creatures like Pokémon characters and long-extinct animals for the interest of people and of children in particular. It is necessary to work on integrating these projects into the education system, into popularizing science, and into gathering high-quality data.

### Ethics Statement

Birds and their nests were not manipulated. Camera monitoring of birds has met relevant national and international guidelines.

## Supporting Information

S1 Fig The wooden structure of a Smart Nest Box for Tengmalm’s owl nesting, built by students in the higher classes of a vocational training school. (TIF)

S1 Text The method for collecting the data on the children’s learning performance. (DOCX)

S2 Text The method for collecting the data on bird nesting. (DOCX)

S1 Data The list of institutions, including addresses, that were registered as participants in the project during the period April—July 2016. (XLSX)

S2 Data Raw data for the analyses on the children’s learning performance. (XLSX)

S3 Data The list of visitors to the websites of Birdsonline.cz. Total numbers and the proportions of visits from each state, together with the mean number of visited pages per visit and the mean duration of a visit.
(XLSX)

S4 Data Raw data for Fig 3. (XLSX)

S1 Video An original video containing unique biological information on great tits, Eurasian tree sparrows, common starlings, pine martens, and Tengmalm’s owls. (MP4)

## Abbreviations

CS: Citizen Science
NGO: nongovernmental organization
SNBox: Smart Nest Box
