# Supplementary material for: A Webcast of Bird Nesting as a State-of-the-Art Citizen Science
Source: PLoS Biol. 2017 Jan 6;15(1):e2001132. doi: 10.1371/journal.pbio.2001132 (PMC5217862; doi:10.1371/journal.pbio.2001132)
Supplement: S1 Text — (DOCX) [file pbio.2001132.s002.docx]

**S1 Text**

We collected evidence on biological learning from 53 children (26 children aged 9, and 27 children aged 10). The children were given a written multiple choice test (they were asked to answer a, b, c, or d). There were questions about identifying bird species, the structure of the nest material, the duration of the breeding period, and the structure of the birds’ diet. The first test was administered two months before the webcast of bird nesting was available, and the second test was administered two months after the bird nesting webcast came into operation. Each child was identified by a specific code number (1-53). The data was tested using the McNemar test based on paired nominal data.
