# Supplementary material for: A Webcast of Bird Nesting as a State-of-the-Art Citizen Science
Source: PLoS Biol. 2017 Jan 6;15(1):e2001132. doi: 10.1371/journal.pbio.2001132 (PMC5217862; doi:10.1371/journal.pbio.2001132)
Supplement: S2 Text — (DOCX) [file pbio.2001132.s003.docx]

**S2 Text**

The data in Fig 3 was collected in the nest of a great tit (*Parus major*) located in the garden of a basic school in Náchod (50°25´N, 16°9‘E). Bird nesting was monitored continually from April 19 to May 23, using Smart Nest Box (SNBox). The SNBox was designed as a regular nest box augmented with additional space for embedding technical components, in particular a camera, a control board, an event detector, and auxiliary sensors (for recording the light intensity and the temperature outside the SNBox, and for recording the temperature inside the SNBox). The camera was activated by an interruption to the event detector located in the nest opening. The camera then operated for 30 s. Software specially developed for central management of all devices stored each video record (for details, see [[1](#_ENREF_1)]). After all data had been collected in the field, bachelor students (from the Czech University of Life Sciences in Prague) extracted biological information from all video records. In particular, they checked the time when each bird entered the SNBox (*n* = 5218). They also recorded whether the bird parent came with food (*n* = 3268), the type of diet (*n* = 2709) and whether the parent removed the droppings of the nestlings from the nest (*n* = 742).

Reference:

1. Zárybnická M, Kubizňák P, Šindelář J, Hlaváč V. Smart nest box: a tool and methodology for monitoring of cavity-dwelling animals. Methods Ecol Evol. 2016; 7: 483-492.
